# Relationships Between Childhood Health Experience and Depression Among Older People: Evidence From China

**DOI:** 10.3389/fpsyg.2021.744865

**Published:** 2021-12-03

**Authors:** Min Yao

**Affiliations:** School of Marxism, Dalian University of Technology, Dalian, China

**Keywords:** childhood health experience, depression, CHARLS, aging, mental health

## Abstract

The assessment of childhood health experience helps to identify the risk of depression among older people. Poor childhood experience is generally associated with depression in adulthood. However, whether such association can be extended to older people’ life remains unclear. The history of parental mental health was obtained from 2014 CHARLS Wave 3 (Life History Survey) data while other data from 2011 CHARLS Wave 1 baseline data. The study involves 4,306 respondents. The depression was assessed by the Chinese version of Center for Epidemiologic Studies Depression scales (CES-D) using logistic regression model. More than 40% of older people suffered from depression, 25% of whom experienced poor childhood self-reported health. Nearly 20% of their mothers and more than 10% of their fathers had a history of poor mental health. Poor childhood health experiences have shown to be associated with higher odds of depression (good self-reported health OR: 0.732, *p* = 0.000, 95% CI: 0.633–0.847; poor mother’s mental health OR: 1.391, *p* = 0.001, 95% CI: 1.138–1.699; poor father’s mental health OR: 1.457, *p* = 0.003, 95% CI: 1.141–1.862). There is a high rate of depression among the older adults in China. In China, older people with poor childhood health experiences are more likely to suffer from depression.

## Introduction

Depression is a common psychiatric disorder among middle-aged and older adults people, affecting an estimated 322 million people worldwide (World Health Organization, 2017). Depression is accompanied with substance abuse (Hjorthøj et al., 2015), chronic pain (Gadermann et al., 2012), impaired quality of life (Ibrahim et al., 2013), reduced life expectancy, and increased risk of suicide (Laursen et al., 2016). Depression is a serious public health issue in both developed and developing countries. Moreover, given the rapid rate of aging worldwide, late-life depression has become a critical concern around the world (Moussavi et al., 2007; Gertner et al., 2017), with its prevalence rising from 4.7% to 16% (Blazer, 2003). According to data from baseline data of the China Health and Retirement Longitudinal Study (CHARLS), 30% of men and 45% of women aged 45 and above in China suffered from depression (Lei et al., 2014), which indicates that depression has become a prominent problem in China. It is suggested that the timely identification of people at high risk of depression helps doctors diagnose the disorder earlier (Cairns et al., 2014). Therefore, the early detection of depression poses a challenge for health improvement.

However, early detection of depression is inseparable from consideration of childhood experiences (Colman and Ataullahjan, 2010), because depression is jointly caused by biological, psychological, and social factors (Urrea and Pedraza, 2000). The retrospective and prospective studies have demonstrated that childhood experiences are related to higher prevalence of late-life depression (Betts, 2014). Furthermore, the life course indicates that early life experiences will produce an enduring impact on the consequences throughout the whole life course (Elder, 1998, 2018; Zheng and Hu, 2018).

Childhood experiences describes experiences which are out of the child’s control such as loss of a parent (Tiet et al., 1998). Childhood is a critical and sensitive period in determining an individual’s later health. Childhood experiences are often linked to physical abuse, sexual abuse, poverty or household dysfunction. Evidences suggest that childhood experiences have accumulative effects on an individual’s health outcomes (Scott et al., 2012). Childhood may shape neurobiological and immunes system development and these changes may persist over the whole life course (Teicher et al., 2003). Currently, many researches focus on the relationship between poor childhood experiences and depression (Chapman et al., 2004; Ford et al., 2011; Nakai et al., 2014; Maclean et al., 2016; Björkenstam et al., 2017; Green et al., 2018) and a negative relationship is observed. However, there is still a research gap in this regard. First, most studies have looked at adults, and it remains unclear whether the relationship extends to older adults. Second, existing studies about childhood experiences do not cover childhood health experiences. Currently, most of the literature concerning the association between childhood experiences and depression focus on childhood neighborhood quality, friendship (Chen et al., 2020), early peer relationship (Jiang and Wang, 2020), etc. However, such research does not take the childhood health into consideration. It is known to all that in China, the older people are the target population, born between 1940s and 1960s. What they have in common is that they all experienced a period of poor childhood health as they were born during war, famine and baby boom (Jiang and Wang, 2020). From the life course perspective, their poor childhood health experience could have a non-negligible and enduring impact on their later psychological health (George, 2014). But the relevant research remains unclear. Therefore, in this paper we aim to fill this research gap by exploring the association between childhood health experiences and depression among older people in China. As noted above, the effects of childhood may persist throughout the life course, and we hypothesize that poor health experiences in childhood may negatively affect depression among older Chinese adults.

## Materials and Methods

### Design and Sample

**FIGURE 1:**
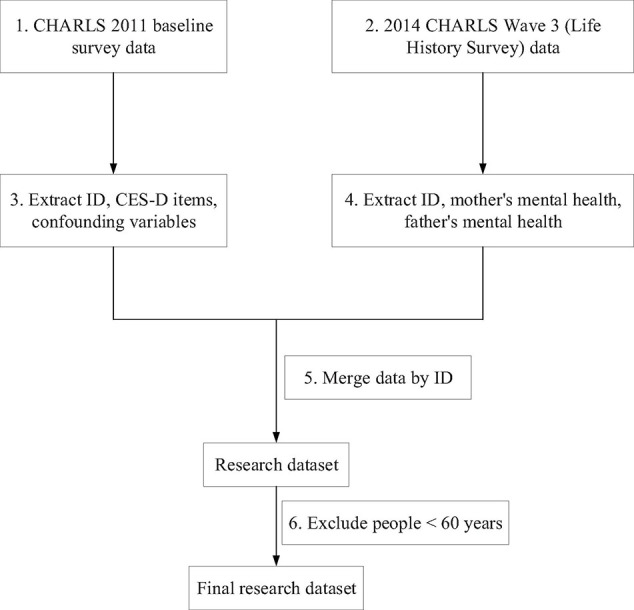
Data processing flow chart.

The China Health and Retirement Longitudinal Study (CHARLS) was conducted to collect a high quality nationally representative sample of Chinese residents aged 45 and above in 2011, which was supported by Peking University, the National Natural Science Foundation of China, the Behavioral and Social Research Division of the National Institute on Aging and the World Bank. The data collection has been approved by the institutional review board at Peking University and the data will be updated annually. About 10,000 households and 17,500 individuals in 150 counties/districts and 450 villages/resident committees were included in the baseline survey. Multi-stage stratified PPS sampling was adopted in CHARLS (Zhao et al., 2014). CHARLS 2011 baseline survey data and 2014 CHARLS Wave 3 (Life History Survey) data were used in this research. Childhood parental health was extracted from 2014 CHARLS Wave3 (Life History Survey) data. All other data were obtained from CHARLS 2011 baseline survey data. People whose age ≥ 60 were included, since 60 years old was the cut-off point between mid-aged people and older people in China. The data processing flow chart is demonstrated in Figure 1.

### Measures

#### Childhood Self-Reported Health

Health is a complex concept and it is difficult to grasp all the relevant aspects. Most of the time, people tend to reflect it on self-reported health status, which is a valuable source of data on various aspects of overall health (Idler and Benyamini, 1997) and is easy to implement. Now it is widely collected in almost all countries. In this research, we adopted childhood self-reported health to reflect the respondents’ heath conditions during childhood. Older people were asked about the following question to assess their childhood self-reported health: how do you evaluate your health during childhood, up to and including age 15? The respondents selecting “excellent,” “very good” or “good” was considered that its childhood self-reported health was good and was coded as 1. Those who answered “fair” or “poor” were considered to have had poor self-reported health as children and were set to 0.

#### Parental Mental Health

Parental mental health has proven to be an important factor affecting the health experience for people’s depression development (Gundel et al., 2018). We included a history of both mother’s and father’s mental health in this paper. The respondents were asked “during the years when you were growing up, did your father/mother show continued signs of sadness or depression that lasted 2 weeks or more.” Those who reported “yes” were considered that his father’s/mother’s mental health was poor and were set as 1 while answer of “no” was set as 0.

#### Health Outcome: Depression

Depression was assessed through the Chinese version of scale items developed by the Center for Epidemiologic Study, with 10 short-form scale items (CESD-10) (Andresen et al., 1994). The CESD-10 contains response options varying from 0 to 4 [0 = Rarely or none of the time (less than 1 day); 1 = Some or a little of the time (1–2 days); 3 = Occasionally or a moderate amount of the time (3–4 days); 4 = Most or all of the time (5–7 days)] (Amtmann et al., 2014). The minimum total score of CESD-10 was 0 and the maximum was 30. The respondents with higher CSED-10 score indicated that he/she had a higher level of depressive symptoms. CES-D score of 10 was the cut-off point to identify whether the respondents experienced significant depressive symptoms. The respondents who were likely to suffer from depression had a CES-D score of ≥ 10 (Lei et al., 2014).

#### Potential Confounding Variables

CHARLS collected data about the respondent’s age, gender, place of residence, marital status and educational level. These variables were potential confounding variables commonly seen in previous studies (Whitaker et al., 2014; Bareis and Mezuk, 2018; Gundel et al., 2018). We included them in this research. In addition, studies have shown that job position (Whitaker et al., 2014) and income (Campbell et al., 2016; Fang and McNeil, 2017) are also potential confounding variables. Therefore, respondent’s retirement status and pension status were included in this research based on the fact that older people’s employment status was dependent on their retirement status and that their income is mainly affected by their pension.

### Analytic Strategy

Continuous variables were displayed through means and standard deviation while categorical variables were described with percentage. A series of logistic regression models were used to assess the association between childhood health experience and depression among Chinese older people: in Model I, we solely accounted for childhood self-reported health; in Model II, we adjusted childhood mother’s mental health separately; Model III was similar to Model II, in which childhood father’s mental health was taken into the model; in Model IV, we included childhood self-reported health, childhood mother’s mental health and childhood father’s mental health simultaneously. The odds ratio (OR) and confidence interval (CI) of 95% were calculated. All models were adjusted for all potential confounding variables mainly including age, gender, place of residence, marital status, educational level, retirement status and pension status. If the *p*-value was less than 0.05, it would be considered as statistically significant. All the work was conducted in Stata, version 13.1 (StataCorp. College Station, TX: Stata Corp LP).

## Results

Descriptive characteristics of the respondents are presented in Table 1. The average age of the population was 67.09 years. Men were slightly older on average than women (Male: 67.11; Female: 67.06). 75% of them experienced good childhood self-reported health. Among males, their fathers (12.3%) were more likely to have a poor mental health than females (11.7%) during their childhood, while among females, their mothers (21.1%) had a higher likelihood to experience bad mental health. In terms of depression prevalence, 40.6% of them suffered from depression. Female older people had a higher depression prevalence than male ones. Depression prevalence for females was 44.5% while for 34.0% for males. Among them, more than 50% still lived in rural areas, nearly 40% retired and about 33% enjoyed pension.

**TABLE 1:** Descriptive characteristics of the participants.

	**Total (4,306)**		**Female (2,151)**		**Male (2,155)**	
**Variables**	**Mean**	**Std**	**Mean**	**Std**	**Mean**	**Std**
**Childhood health experience**						
Self-reported health (good)	75.0		74.5		75.5	
Mother’s mental health (poor)	19.7		21.1		18.3	
Father’s mental health (poor)	12.0		11.7		12.3	
Age	67.09	5.97	67.06	6.12	67.11	5.81
Gender (male)	50.0					
**Place of residence**						
Rural	63.8		62.6		65.0	
**Marital status**						
Married	78.4		71.6		85.2	
Partnered	2.3		2		2.6	
Separated	0.7		0.6		0.7	
Divorced	0.6		0.4		0.8	
Widowed	17.1		25.2		9.0	
Never married	0.8		0.1		1.6	
**Education level**						
Primary school	26.5		17.8		35.2	
Middle school	12.7		8.1		17.4	
High school	5.0		2.7		7.2	
College and above	1.6		0.7		2.6	
Illiteracy	54.1		70.8		37.5	
CES-D score	8.93	6.39	10.11	6.73	7.75	5.81
CES-D score ≥ 10	40.6		49.4		31.9	
**Retirement status**						
Retired	39.3		44.5		34.0	
Pension status						
Received	33.3		32.4		34.2	

Table 2 shows the results of logistic regression analyses. First, we assessed the relationship between childhood self-reported health and depression among older people by Model I. Older people with good self-reported health in childhood significantly had lower likelihood of suffering from depression than those with poor childhood self-reported health as the OR was less than 1 (OR = 0.719^∗∗∗^, 95% CI: 0.622–0.830). Next, we evaluated the association between parental mental health in childhood and depression among older people by Model II and Model III. The results indicate that poor parental mental health is associated with a greater probability of having depression, since “mother’s mental health” had an OR of 1.696^∗∗∗^ (95% CI: 1.449–1.986) and “father’s mental health” had an OR of 1.892^∗∗∗^ (95% CI: 1.559–2.296). Finally, when we put the three childhood health experience factors into Model IV together, these significant relationships still existed.

**TABLE 2:** Associations between childhood self-reported health, parental mental health and depression among Chinese older people.

	**Model I**	**Model II**	**Model III**	**Model IV**
**Childhood health experience**				
Self-reported health (good)	0.719^***^			0.732^***^
	[0.622, 0.830]			[0.633, 0.847]
Mother’s mental health (poor)		1.696^***^		1.391^**^
		[1.449, 1.986]		[1.138, 1.699]
Father’s mental health (poor)			1.892^***^	1.457^**^
			[1.559, 2.296]	[1.141, 1.862]
Age	0.994	0.995	0.994	0.995
	[0.982, 1.006]	[0.983, 1.006]	[0.983, 1.006]	[0.984, 1.007]
Gender	0.539^***^	0.539^***^	0.529^***^	0.533^***^
	[0.469, 0.619]	[0.469, 0.620]	[0.460, 0.608]	[0.463, 0.613]
**Place of residence**				
Rural	1.464^***^	1.440^***^	1.446^***^	1.421^***^
	[1.267, 1.691]	[1.246, 1.664]	[1.251, 1.671]	[1.229, 1.644]
**Marital status**				
Married	0.299^***^	0.278^***^	0.289^***^	0.295^***^
	[0.147, 0.606]	[0.137, 0.564]	[0.142, 0.587]	[0.145, 0.601]
Partnered	0.334^**^	0.305^**^	0.319^**^	0.325^**^
	[0.148, 0.752]	[0.135, 0.688]	[0.141, 0.720]	[0.143, 0.736]
Separated	0.746	0.619	0.636	0.641
	[0.264, 2.112]	[0.218, 1.763]	[0.224, 1.806]	[0.225, 1.828]
Divorced	0.519	0.488	0.515	0.535
	[0.180, 1.497]	[0.169, 1.406]	[0.179, 1.486]	[0.184, 1.550]
Widowed	0.394^*^	0.368^**^	0.381^**^	0.389^*^
	[0.191, 0.812]	[0.178, 0.761]	[0.185, 0.788]	[0.188, 0.806]
**Education level**				
Primary school	0.866	0.892	0.892	0.901
	[0.742, 1.012]	[0.763, 1.042]	[0.764, 1.043]	[0.770, 1.053]
Middle school	0.606^***^	0.631^***^	0.626^***^	0.638^***^
	[0.488, 0.754]	[0.507, 0.785]	[0.504, 0.779]	[0.512, 0.794]
High school	0.471^***^	0.481^***^	0.485^***^	0.490^***^
	[0.331, 0.670]	[0.338, 0.685]	[0.341, 0.690]	[0.344, 0.698]
College	0.251^***^	0.245^***^	0.253^***^	0.246^***^
	[0.118, 0.535]	[0.115, 0.522]	[0.119, 0.538]	[0.116, 0.525]
**Retirement status**				
Retired	0.998	1.003	1.007	1.015
	[0.863, 1.154]	[0.867, 1.160]	[0.871, 1.165]	[0.877, 1.175]
**Public pension status**				
Received	0.796^**^	0.801^**^	0.799^**^	0.803^**^
	[0.695, 0.912]	[0.699, 0.918]	[0.697, 0.916]	[0.700, 0.920]
*R* ^2^	0.0549	0.0589	0.0587	0.0636
*N*	4,306	4,306	4,306	4,306

*Exponentiated coefficients; 95% confidence intervals in brackets.*

*p < 0.05, **p < 0.01, ***p < 0.001.

*Reference level: gender (female); marital status (unmarried); education level (illiteracy); place of residence (urban).*

## Discussion

Former studies concentrate more on the enduring health effect of early childhood adversities, and the enduring health effect of childhood health experience has received little attention. Drawing on the life course theory, this is the first study to assess the enduring health effect of multidimensional childhood health experiences, including childhood self-reported health and parental mental health. Using a representative sample of 4,306 individuals, this study examined whether childhood health experience was associated with depression among older people in China. The results confirm our hypothesis that after adjusting for several confounding variables, both poor childhood self-reported health and poor childhood parental mental health are associated with higher likelihood of suffering from depression among older people. Thus, more attention should be paid to older people who had poor childhood health experience to actively cope with the high prevalence of depression among older people in China. This study is a comprehensive research to explore the childhood health experience factors influencing older people’s depressive symptoms.

Generally speaking, the results of this study support the view of life course theory that early life experiences will produce an enduring impact on the consequences throughout the whole life course (Elder, 1998, 2018; Zheng and Hu, 2018). The findings demonstrate that poor childhood self-reported health experience had a strong positive correlation with depressive symptoms, which is in line with the results of Danese and McEwen (2012). Childhood experiences covered different aspects, such as physical abuse, parental separation, low family socioeconomic status and malnutrition. It is reported that a history of poor childhood experience is a high-risk group with respect to depression in young adulthood (Björkenstam et al., 2017). Our findings are consistent with those of the study on childhood experience, implying that poor childhood experience is correlated with depression. Prior studies looking at the childhood experiences related to mental health also support this relationship. Anda et al. (2002) point out that the number of poor childhood experiences had a graded relationship to depression (Anda et al., 2002). In their study, one of the poor childhood experiences that they used was mentally illness, as an aspect of childhood health. They were assessed for depression in adulthood. Another similar research confirms this negative relationship. Gundel et al. (2018) indicate that children and adolescents with non-affective mental disorders are at substantially increased absolute and relative risk of developing depression in young adulthood. And an increased relative risk for depression in children with anxiety disorders has also been observed (Copeland et al., 2013). The results of poor childhood self-reported health in this study are consistent with theirs. But childhood self-reported health reflects a more comprehensive health status in childhood than mental health used in other studies, and we extended the negative relationship to older people, which was more specific. This can be explained by the following reasons based on the life course perspectives. Many literatures have suggested that nutritional adversity in childhood will have a lasting impact on people’s health throughout the life course (Gluckman et al., 2005; Kesternich et al., 2014). As mentioned earlier, older people suffered from malnutrition in childhood because they were born during the war, the famine and the baby boom (Jiang and Wang, 2020). The consequences caused by poor childhood health may accumulate over time and can finally lead to chronic diseases later in life, while chronic diseases may increase risk of depression.

History of parental health status is an important childhood experience for everyone. Our results demonstrate that children whose parents suffer from mental health problems are associated with a higher risk of depression in the older age. Previous studies examined the impact of different factors such as psychopathology, morbidity, and mortality on the offspring of parents with depression from birth to adulthood, and found that people whose parents had poor mental health status were more likely to have depression (Weissman et al., 1997, 2016, 2006). Chapman et al. (2018) indicate that exposure to mental illness during childhood has an effect on the risk of depressive disorders. Prior studies have shown that history of adverse parental status contributes to depression (Björkenstam et al., 2017). It is suggested that parental death, parental substance abuse, substantial parental criminality, parental psychiatric morbidity and parental separation help to predict depression in early adulthood (Green et al., 2010; Sareen et al., 2013; Björkenstam et al., 2017; Dahl et al., 2018). In particular, parental psychiatric morbidity has been reported to be a key factor for depression (Chapman et al., 2018). In this research, we used history of parental mental health status as an indicator for childhood health experience. Our results are in line with theirs. We found that individuals who had history of poor parental mental health were at a high risk of depression, as suggested by previous literature (Björkenstam et al., 2017). This can also be rationally explained by the life course theory that parental mental health has an enduring effect on offspring’s mental health outcomes (Ferraro and Shippee, 2009). When living with their parents with poor mental health status, people in their childhood were easily affected by negative emotions, such as depression, anxiety and fear. Additionally, they could not receive adequate health care and emotional support from their parents. Thus, they were surrounded with a poor health status atmosphere. The long-term surroundings will promote their depressive symptoms development, which can lead to life-long consequences.

This study has the following limitations. First, we relied on self-reported data on childhood health experience. However, the low prevalence of adverse childhood health experiences in this population sample may reflect recall bias. A common fact is that most of the population in this sample was born 60 years ago while they possibly lived through the Great Chinese Famine at that time or they may be malnourished. And at that time the death rate was very high (Feng and Johansson, 2018). However, only a quarter of them reported that their childhood health was poor. Therefore, our results may be underestimated. Another drawback is that we only used CES-D to assess depression. Although CES-D has been widely used to assess depression, it is not a golden rule to diagnose depression and CES-D may result in an underestimation or overestimation for depressive symptoms. Finally, we only included three childhood health indicators in our research because we hoped to conduct our research from the perspective of childhood health experience. Other childhood experiences like childhood neighborhood quality and relationships with parents may also influence depression among older people. Further research is needed to explore the impact of other potential factors on depression.

## Conclusion

In conclusion, we find that having poor childhood health experience is associated with depression in older people. The results show that individuals with poor childhood health experience are at high risk of depression in older life. Given the importance of childhood, early and enough support for older people of having poor childhood health is important to improve their mental health. And early preventive interventions should invest in children with a history of poor childhood health experience.

## Data Availability Statement

Publicly available datasets were analyzed in this study. This data can be found here: charls.pku.edu.cn/.

## Ethics Statement

The study was reviewed and approved by the Institutional Review Board of Peking University with ethical approval no. (IRB00001052-11014). Written informed consent was obtained from the individual(s) for the publication of any potentially identifiable images or data included in this article.

## Author Contributions

The author confirms being the sole contributor of this work and has approved it for publication.

## Conflict of Interest

The author declares that the research was conducted in the absence of any commercial or financial relationships that could be construed as a potential conflict of interest.

## Publisher’s Note

All claims expressed in this article are solely those of the authors and do not necessarily represent those of their affiliated organizations, or those of the publisher, the editors and the reviewers. Any product that may be evaluated in this article, or claim that may be made by its manufacturer, is not guaranteed or endorsed by the publisher.
